# Post-transplant inflow modulation for early allograft dysfunction after living donor liver transplantation

**DOI:** 10.1186/s40792-020-00897-8

**Published:** 2020-07-08

**Authors:** Mohamed Elshawy, Takeo Toshima, Yoshiki Asayama, Yuichiro Kubo, Shinichiro Ikeda, Toru Ikegami, Shingo Arakaki, Tomoharu Yoshizumi, Masaki Mori

**Affiliations:** 1 Department of Surgery and Science, Graduate School of Medical Sciences, Kyushu University, 3-1-1 Maidashi, Higashi-ku, Fukuoka, 812-8582 Japan; 2 Department of General Surgery, Faculty of Medicine, Ain Shams University, Cairo, Egypt; 3 Department of Clinical Radiology, Graduate School of Medical Sciences, Kyushu University, Fukuoka, Japan; 4 Department of Infectious, Respiratory, and Digestive Medicine, Graduate School of Medicine, University of the Ryukyus, Nakagami, Okinawa, Japan

**Keywords:** Liver transplantation, Portal flow, Modulation, Graft dysfunction, Small-for-size syndrome, Splenic artery, Embolization

## Abstract

**Background:**

To treat small-for-size syndrome (SFSS) after living donor liver transplantation (LDLT), many procedures were described for portal flow modulation before, during, or after transplantation. The selection of the procedure as well as the best timing remains controversial.

**Case presentation:**

A 43-year-old female with end-stage liver disease underwent LDLT with extended left with caudate lobe graft from her donor who was her 41-year-old brother (graft volume/standard liver volume (GV/SLV), 35.7%; graft to recipient weight ratio (GRWR), 0.67%). During the surgery, splenectomy could not be performed owing to severe peri-splenic adhesions to avoid the ruined bleedings. The splenic artery ligation was not also completely done because it was dorsal to the pancreas and difficult to be approached. Finally, adequate portal vein (PV) inflow was confirmed after portal venous thrombectomy. As having post-transplant optional procedures that are accessible for PV flow modulation, any other procedures for PV modulation during LDLT were not done until the postoperative assessment of the graft function and PV flow for possible postoperative modulation of the portal flow accordingly. Postoperative PV flow kept as high as 30 cm/s. By the end of the 1st week, there was a progressive deterioration of the total bilirubin profile (peak as 19.4 mg/dL) and ascitic fluid amount exceeded 1000 mL/day. Therefore, splenic artery embolization was done effectively and safely on the 10th postoperative day (POD) to reverse early allograft dysfunction as PV flow significantly decreased to keep within 20 cm/s and serum total bilirubin levels gradually declined with decreased amounts of ascites below 500 mL on POD 11 and thereafter. The patient was discharged on POD 28 with good condition.

**Conclusions:**

SFSS can be prevented or reversed by the portal inflow modulation, even by post-transplant procedure. This case emphasizes that keeping accessible angiographic treatment options for PV modulation, such as splenic artery embolization, after LDLT is quite feasible.

## Introduction

Living donor liver transplantation (LDLT) is a commonly used treatment option for patients with end-stage liver disease, particularly in eastern countries [1, 2]. Small-for-size syndrome (SFSS) can lead to serious early graft dysfunction after LDLT. The main clinical presentation is cholestasis, prolonged ascites, coagulopathy, and encephalopathy [3]. Extra small graft, portal hyperperfusion, severe portal hypertension, and venous outflow obstruction are the main underlying causes of SFSS [4]. To prevent or reverse this drastic complication, many procedures were described for portal flow modulation before, during, or after the transplant surgery; however, the selection of the procedure as well as the best timing is still controversial [5–9].

Herein, we report a case of LDLT recipient who developed early graft dysfunction after LDLT and SFSS with evident high portal flow. Postoperative splenic artery embolization (SAE) was done as a post-transplant portal inflow modulation, and SFSS was successfully treated.

## Case presentation

The patient was a 43-year-old female with end-stage liver disease secondary to HCV hepatitis. Her weight was 60 kg and her body mass index was 24.3 kg/m^2^. Her blood type was O Rh (+). Preoperative assessment reveals the Child-Pugh score was grade C as 13 pts and the model for end-stage liver disease score was high as 19. Radiological evaluation revealed partial PV thrombosis in the main trunk. She underwent partial SAE 8 years ago and umbilical hernia repair. Her donor was her 41-year-old brother who weighted 79 kg and body mass index was 26.7 kg/m^2^. His blood type was B Rh (+). Preoperative 3-dimensional volumetry revealed that the extended left with caudate lobe graft volume was 555 mL, which was 49.2% of the recipient standard liver volume (SLV). The donor and recipient have incompatible blood types, so the recipient was subjected to preoperative rituximab protocol.

The patient underwent LDLT using extended left with caudate lobe graft. The actual graft weighted 467 g (402 g after UW reperfusion) of which GV/SLV was 35.7% and GRWR was 0.67%. It had middle and left hepatic veins, left hepatic artery, left PV, and left hepatic duct. Upon laparotomy, there was 10,500 mL of ascites as well as perihepatic adhesions and periumbilical adhesions. On laparotomy, portal venous pressure (PVP) monitoring was 22 mmHg. The splenic artery was dorsal to the pancreas and difficult to be approached, and finally, the upper pole branch of the splenic artery was identified and ligated at the level of the distal pancreas, by which only the upper pole of the spleen turned pale. Splenectomy could not be performed due to severe peri-splenic adhesions to avoid the ruined bleedings. After PV thrombectomy was done, adequate PV inflow was not confirmed until left gastric vessel ligation was performed. After graft reperfusion, PVP was 20 mmHg but PV flow was relatively low, 520 mL/min. At this time, we choose not to ligate the other branches of the splenic artery nor the lienorenal shunt until postoperative assessment of the graft function and the PV flow for possible postoperative modulation and boost of the portal flow accordingly. The point is that safe angiographic procedures for portal flow modulation are accessible postoperatively, such as splenic artery embolization for portal decompression and balloon-occluded retrograde transvenous obliteration (BRTO) for boosting portal flow.

**Fig. 1:**
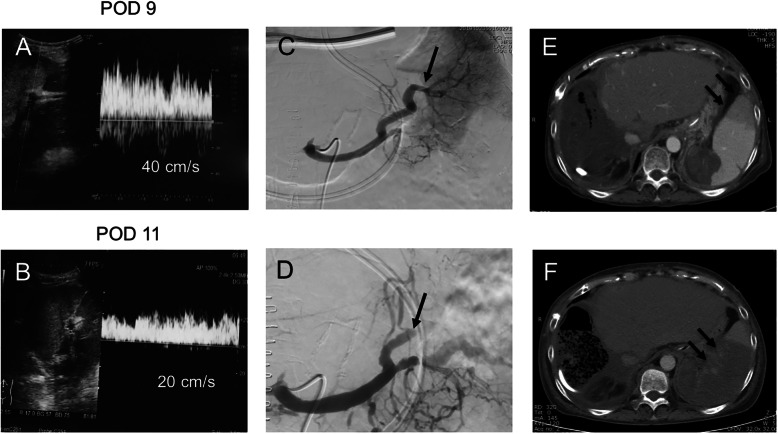
Postoperative assessment of portal flow and dynamic CT image before (**a**, **c**, **e**) and after (**b**, **d**, **f**) splenic artery embolization. **a** Portal flow at the umbilical portion on postoperative day (POD) 9. **b** Portal flow on POD 11 at the same branch of the portal vein, which had decreased to half of that on POD 9. **c** Splenic arterial overflow with splenomegaly showed enhanced flow on injection of the contrast. Arrow, the main branch of the splenic artery. **d** The main branch of the splenic artery was completely occluded by trans-catheter coil embolization on POD 10. Arrow, the main branch of the splenic artery. **e** Dynamic computed tomography (CT) before splenic artery embolization showed only the upper pole of the spleen turns pale by the ligation. Arrow, paled lesion of the spleen. The lower pole has lost the arterial flow due to previous partial splenic artery embolization. **f** Dynamic CT after splenic artery embolization showed most of the spleen lost the arterial flow. Arrow, paled lesion of the spleen. POD, postoperative day; CT, computed tomography

**Fig. 2:**
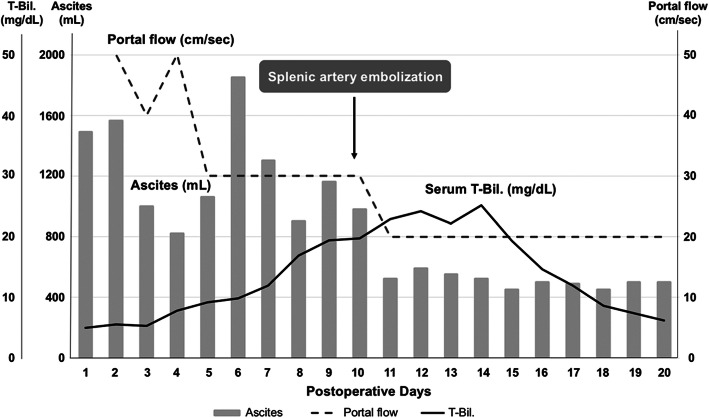
Clinical course and laboratory workup after living donor liver transplantation pre- and post-splenic artery embolization

After LDLT, routine abdominal ultrasound screening revealed that portal flow was 60 cm/s on POD 1, then it kept as high as 30 cm/s along the 1st week (Fig. 1a). Ascites volume was initially below 1000 mL/day till 6th POD, however, increased markedly thereafter. Serum bilirubin levels showed a stepwise gradual increase that reaches 19.4 mg/dL on POD 9 (Fig. 2). Platelet counts were lowest (42 × 10^3^/μL) by POD 8 and INR levels kept below 1.5 along the whole clinical course. Considering this relatively insufficient graft volume along with the clinical and laboratory parameters suggestive of SFSS, then we decided to do splenic artery embolization (SAE) for portal decompression. The patient underwent arterial splenic angiography on POD 10 via percutaneous trans-femoral Seldinger’s technique. After angiographic assessment, partial SAE occluding the main branch of the splenic artery was achieved by trans-catheter coil embolization (Fig. 1c, d). Dynamic computed tomography comparing the spleen pre- and postembolization are also shown (Fig. 1e, f).

After SAE, PV flow significantly decreased to keep within 20 cm/s by US study (Fig. 1b). Additionally, serum total bilirubin levels gradually declined and ascites amounts decreased below 400 mL (Fig. 2), and then, the abdominal drain was removed on POD 21. Platelet counts showed a stepwise increase thereafter. The patient was discharged on POD 28 in good condition without any infectious complications.

## Discussion

SFSS is a catastrophic complication that can lead to graft failure and retransplantation [10]. The classic postoperative course is graft dysfunction within the first 2 post-transplant weeks with two of the following: prolonged functional cholestasis (serum total bilirubin levels > 5.0 mg/dL), intractable ascites (1000 mL/day), and/or coagulopathy (INR > 2) [10–13]. SFSS should be differentiated from graft dysfunction due to other pathological abnormalities [14]. For instance, technical (e.g., arterial or portal occlusion, venous outflow congestion, bile leak), immunological (e.g., acute rejection after LDLT), and infectious (e.g., cholangitis, sepsis) abnormalities can lead to graft dysfunction and overlapped clinical presentation [11, 12]. Moreover, recent studies have documented that GRWR less than 0.8% or GV/SLV less than 40% do not necessarily lead to SFSS [15–19]. Indeed, multiple variables are attributed including preoperative recipient disease severity, donor age, portal pressure and/or flow, graft type, and graft regeneration [3, 17, 20, 21].

This case presents a patient who underwent ABOi-LDLT using extended left with caudate lobe graft that was relatively insufficient as the GV/SLV 35.7% and at high risk of SFSS after the surgery. Early graft dysfunction was imminent giving progressive hyperbilirubinemia and intractable ascites. Radiological evaluation excluded technical problems that may be the cause of the clinical presentation. Liver transaminases were initially high; however, along the 1st postoperative week, a progressive decline towards near normal levels precludes possible acute rejection. Therefore, along with a high postoperative portal flow, SFSS diagnosis was highly suspected. Coagulopathy, represented by high prothrombin time and INR, may be along the clinical presentation of SFSS [10, 14]. However, in their series, Gruttadauria et al. reported that coagulopathy was not a reliable indicator of SFSS [7]. In the present case, though INR values showed a slight increase before splenic artery embolization SAE, they were kept below 1.5 before as well as after portal flow modulation.

High post-perfusion PVP had been reported to negatively impact graft outcome [22, 23]. In the setting of small-for-size graft after LDLT, persistent elevation of PVP causes direct hepatocyte injury due to sinusoidal shear stress, congestion, hemorrhage, and endothelial activation [24–26]. Indeed, secondary ischemic changes occur due to adaptive hepatic artery vasoconstriction [27]. A key management strategy is portal flow modulation with partially diverting portal flow via portosystemic shunt [28] and/or portal decompression by splenectomy [3, 29, 30], splenic artery ligation [29, 31], or splenic artery embolization [7, 32, 33]. Following LDLT, maintaining adequate portal inflow is crucial for boosting graft regeneration [34]. In the setting of portal hypertensive liver cirrhosis, high sinusoidal resistance diverts portal flow, via portosystemic collaterals, which may jeopardize the graft [35]. Portal steal phenomenon can also occur due to hepatofugal diversion of portal flow through major (> 1 cm) portosystemic shunts [36, 37] with subsequent graft ischemic injury and possible post-transplantation PV thrombosis [28, 38, 39].

We [40, 41] previously reported the beneficial effects of simultaneous splenectomy for recipients with PVP more than 15 mmHg following graft reperfusion. In addition, we previously described that en bloc division of large portosystemic shunts along with splenectomy should simplify and normalize portal hemodynamics with the best graft outcome [42, 43]. On the contrary, the Tokyo University group reported that splenectomy was an independent predictor for postoperative hemorrhage and sepsis; hence, they restricted simultaneous splenectomy in strictly indicated recipients [44]. Moon et al. compared simultaneous splenectomy to an innovative technique, splenic devascularization in adult LDLT. A higher incidence of procedural-related complications was observed in the splenectomy group, as pancreatic fistula, abscess, and hemorrhage, though, did not reach statistical significance [45]. In the setting of LDLT, simultaneous splenectomy often leads to higher morbidity. In the present case, extensive peri-splenic adhesions put the patient at high risk for simultaneous splenectomy. Attempts to ligate the main splenic artery also make the patient at high risk for distal pancreatic injury which can lead to post-transplant catastrophic pancreatic complications. Moreover, following PV thrombectomy and reperfusion, as we previously described, PVP was relatively adequate [46]; however, PV flow was 520 mL/s. As we have the postoperative optional procedures that are accessible for portal flow modulation, then additional intraoperative procedures were not performed during LDLT until postoperative assessment of graft function and PV flow. For instance, SAE can be an alternative for portal decompression [7, 32, 33] and BRTO can be used for boosting portal hypoperfusion salvaging against portal steal preventing graft ischemia [47]. Postoperative portal hemodynamics were disturbed as PV inflow kept high along with marked intractable ascites and serum hyperbilirubinemia, then postoperative SFSS ensued. SAE effectively reversed early allograft dysfunction and impending SFSS.

Previous reports have already pointed to SAE for post-transplant portal modulation. For instance, Gruttadauria et al. [7] reported a series of six patients; however, all were right lobe graft with mean GRWR 1.282 ± 0.276%. Although the clinical presentation was nearly similar to this report, they did not refer to portal pressure nor flow in the peritransplant management. In the present case, the graft of extended left with caudate lobe was low as GRWR 0.67%. After graft reperfusion, finally, PVP was relatively high as 20 mmHg, while PV flow was relatively low as 520 mL/min. Therefore, we choose to monitor portal hemodynamics and decide further management giving accessible angiographic options postoperatively.

Reported rates of complications after SAE, as splenic abscesses, splenic infarction, infections, bleeding, pancreatitis, or postembolization syndrome (abdominal pain, fever, and increased levels of pancreatic enzymes), widely vary [48, 49]. In the setting of post-transplant SFSS, SAE is a safe and feasible option. Among 54 patients who underwent post-transplant SAE, Presser et al. [32] reported one case of post-splenectomy syndrome (transient leukocytosis and fever which resolve spontaneously shortly after SAE). The present case did not develop any post SAE complications.

Giving post-transplantation portal modulation dilemma [6, 9, 32, 50], postoperative portal modulation can be an alternative to high-risk intraoperative procedures. As portal hyperperfusion is contributed by splenic flow [7], an angiographic intervention can aid and may be more effective in post-transplant portal inflow modulation when necessary. For instance, the present case emphasizes that in the setting of relatively small-for-size graft, post-transplant portal decompression with splenic artery embolization can safely rescue from impending SFSS in case of increased portal inflow postoperatively.

## Conclusion

We report here the case of a patient who recovered from SFSS by the postoperative portal flow modulation by splenic artery embolization. The present case suggests that keeping the accessible angiographic treatment options for PV modulation after LDLT is quite feasible.

## Data Availability

All data are available on request.

## Abbreviations

BRTO: Balloon-occluded retrograde transvenous obliteration
GRWR: Graft to recipient weight ratio
GV/SLV: Graft volume/standard liver volume
LDLT: Living donor liver transplantation
PV: Portal vein
PVP: Portal venous pressure
POD: Postoperative day
SFSS: Small-for-size syndrome
SAE: Splenic artery embolization
