# Correction to: Post-transplant inflow modulation for early allograft dysfunction after living donor liver transplantation

**DOI:** 10.1186/s40792-020-00958-y

**Published:** 2020-08-04

**Authors:** Mohamed Elshawy, Takeo Toshima, Yoshiki Asayama, Yuichiro Kubo, Shinichiro Ikeda, Toru Ikegami, Shingo Arakaki, Tomoharu Yoshizumi, Masaki Mori

**Affiliations:** 1 Department of Surgery and Science, Graduate School of Medical Sciences, Kyushu University, 3-1-1 Maidashi, Higashi-ku, Fukuoka, 812-8582 Japan; 2 Department of General Surgery, Faculty of Medicine, Ain Shams University, Cairo, Egypt; 3 Department of Clinical Radiology, Graduate School of Medical Sciences, Kyushu University, Fukuoka, Japan; 4 Department of Infectious, Respiratory, and Digestive Medicine, Graduate School of Medicine, University of the Ryukyus, Nakagami, Okinawa, Japan

**Correction to: Surg Case Rep 6, 164 (2020)**

**https://doi.org/10.1186/s40792-020-00897-8**

Following publication of the original article [1], the authors would like to correct the content under **Acknowledgement**.

The content currently reads:

We would like to thank Edanz Company for the English language editing.

The content should be changed to:

M.E would like to thank the Ministry of Higher Education and Scientific Research, Cultural Affairs and Missions Sector, Egypt for the educational scholarship.
